# Selective genotyping to implement genomic selection in beef cattle breeding

**DOI:** 10.3389/fgene.2023.1083106

**Published:** 2023-03-17

**Authors:** Maryam Esrafili Taze Kand Mohammaddiyeh, Seyed Abbas Rafat, Jalil Shodja, Arash Javanmard, Hadi Esfandyari

**Affiliations:** ^1^ Department of Animal Sciences, University of Tabriz, Tabriz, Iran; ^2^ Norwegian Beef Cattle Organizations, TYR, Hamar, Norway

**Keywords:** beef, genomic estimated breeding value, pedigree, SSGblup, meta-founder

## Abstract

Genomic selection (GS) plays an essential role in livestock genetic improvement programs. In dairy cattle, the method is already a recognized tool to estimate the breeding values of young animals and reduce generation intervals. Due to the different breeding structures of beef cattle, the implementation of GS is still a challenge and has been adopted to a much lesser extent than dairy cattle. This study aimed to evaluate genotyping strategies in terms of prediction accuracy as the first step in the implementation of GS in beef while some restrictions were assumed for the availability of phenotypic and genomic information. For this purpose, a multi-breed population of beef cattle was simulated by imitating the practical system of beef cattle genetic evaluation. Four genotyping scenarios were compared to traditional pedigree-based evaluation. Results showed an improvement in prediction accuracy, albeit a limited number of animals being genotyped (i.e., 3% of total animals in genetic evaluation). The comparison of genotyping scenarios revealed that selective genotyping should be on animals from both ancestral and younger generations. In addition, as genetic evaluation in practice covers traits that are expressed in either sex, it is recommended that genotyping covers animals from both sexes.

## 1 Introduction

In livestock breeding programs, genomic selection (GS) is a method that uses genomic information to estimate breeding values and rank selection candidates. GS has shaped modern breeding programs and contributed substantially to the increase of genetic progress for various economically important traits, especially in dairy cattle (VanRaden et al., 2009; Meuwissen et al., 2016). The advantages of GS over traditional selection include; shorter generation intervals, increased selection intensity, greater selection accuracies, not limited to sex, and can be generalized to any trait that is recorded in the reference population (Schaeffer, 2006; Aguilar et al., 2010).

Genomic selection has a high potential for improving the genetic gain in beef cattle because reproduction, health, growth rate, meat quality, and feed efficiency are vital traits that contribute to the profitability of this industry, which are difficult and expensive to measure routinely (Van Eenennaam et al., 2011; Montaldo et al., 2012; Hayes et al., 2013). However, the accuracies of genomic breeding values for economic traits in beef cattle are low to moderate (Saatchi et al., 2011; Van Eenennaam et al., 2014). This is for two possible reasons: i) the reference populations that have been assembled for beef cattle are generally smaller than those for dairy cattle, and there are fewer sires with highly accurate progeny tests in comparison with dairy cattle; and ii) unlike dairy cattle, where populations around the world are dominated by just a couple of breeds, there are numerous breeds of importance and even two subspecies (Bos *taurus* and B. *indicus*) in the beef industry (Hayes et al., 2013).

Genomic selection in beef cattle was first performed based on pseudo-data with multiple-step methods, such as estimated breeding value (EBV) or daughter yield deviation (VanRaden et al., 2009). This method needs many animals (hundreds of thousands) to be genotyped and have phenotypic measurements for the trait of interest to serve as the reference population. The reference population also needs to be updated, i.e., new animals with both phenotype and genotype need to be added. Although the multiple-step method is practical, it rests on several assumptions that are not met in all situations; for instance, it is impossible to genotype all animals. Also, the predicted accuracy using the multistep procedure is lower when compared to single-step BLUP. Also, the large number of breeds and crossbreds, poor extent of phenotyping, limited use of artificial insemination, less advanced structures and breeding programs, low number of offspring per female, incomplete relationships between identical traits in different countries, and limited data recording on economically important traits have resulted in limited adoption of GS in beef cattle (Goddard, 2009; Johnston et al., 2012; Van Eenennaam et al., 2014). Despite these difficulties, results of applying GS have been reported in some studies (Hayes et al., 2019; Wang et al., 2019; Zhu et al., 2019). All studies reported the benefits of applying GS in beef cattle and showed that GS could be a practical alternative to traditional selection approaches. Due to the mentioned limitations, the single-step genomic best linear unbiased prediction (ssGBLUP) method that combines all types of information (phenotype records, pedigree, genotypes) seems to work best in practical genetic evaluation in beef cattle. The main benefit of this method is that all animals in evaluation can get genomic-enhanced breeding values, even if not all have been genotyped (Misztal et al., 2009; Legarra et al., 2014).

Berry et al., 2016 reviewed prediction accuracy with the use of genomic data for some traits in beef cattle. However, guidelines to implement the method in practice are lacking. The main challenge in the implementation of GS for a breeding organization would be which and how many animals to be genotyped as the initial step. Even though, the cost of genotyping is not an obstacle nowadays, but contrary to dairy, the beef industry is much smaller, and genetic evaluation is performed on a much smaller scale in most countries. This is because beef production is highly influenced by the dairy sector with calves and cattle not required for dairy products being fattened to produce meat (Deblitz, 2008). Smaller industries can easily translate to the fact that breed associations and companies have much fewer resources to spend on genotyping. In the ideal situation, there may be possibilities to spend some funding on genotyping of the semen sires that are in service. However, a large proportion of genotyping costs for younger animals would be paid by the farms which traditionally are slower adopters of technology than dairy farmers. This could be due to a multitude of reasons, including the lower business margin.

While genotyping of the ancestral sires in a sense that they have contributed much more than their contemporaries to the current generations seems logical, however, sustainable genetic gain in a breeding scheme needs accurate selection in younger generations as well. In addition, similar to dairy cattle, genetic progress in a breeding scheme in beef is not only driven by bulls but also depends on the superiority of the dams of the candidates. Based on this, we hypothesized that prediction accuracy in selection candidates might differ when genotyping is only on ancestors, younger generation, and is restricted for males or females. Thus, the main aim of this study was to evaluate genotyping strategies in terms of prediction accuracy as the first step in the implementation of GS in beef. In particular, the goal was to compare and to contrast the importance of genotyping the ancestors and distributing the genotyped individuals for the two sexes on the predicting accuracy. The study was conducted under the assumption that a number of animals being genotyped is the main constraint.

## 2 Materials and methods

### 2.1 Definition of the population structure

Using QMSim software, a historical population of beef cattle was simulated based on the forward-in-time process (Sargolzaei and Schenkel, 2009). In total, 2020 generations were considered for the historical population. For the first 1,000 generations, the population size ( n = 1,000) was constant and gradually decreased to 200 individuals to generate linkage disequilibrium (LD) during generations 1,001 to 2020.

In the second step, to enlarge the base population, 100 Founder males and 100 Founder females were selected randomly from the last generation of the historical population (Expanded generations) and were mated randomly for another eight generations. In the third step (Breed formation), five random samples, as the base for five breeds (A-E), were randomly chosen from the last generation of the previous step. In this step, mating and selection were also random within each breed, producing two offspring per dam for 30 generations. In the last step (step 4), population structure was simulated to mimic the production and genetic evaluation system in practice such that parameters were chosen to be realistic five breeds with different sizes were simulated for 15 generations. Selection in all breeds was based on EBVs using pedigree-based BLUP, and the culling of animals was based on age. It was assumed that pedigree was available for all breeds without error, and base animals in this step were considered as meta founders (i.e., one meta-founder per breed). Sire and dam replacement ratio was different across breeds (Table 1).

**TABLE 1:** Parameters of the simulation process.

Population structure	
Step 1: Historical generations (HG)	
Number of generations phase 1	1,000
Size	1,000
Number of generations phase 2	200
Size	2020
Step 2: Expanded generations (EG)	
Number of founder males from HG	100
Number of founder females from HG	100
Number of generations	8
Number of offspring per dam	5
Selection and mating	Random
Step 3: Breed formation (BF)	
Number of males/females from BF for all 5 breeds	100/100
Number of generations	30
Number of offspring per dam	2
Selection and mating	Random
Step 4: Breeds A, B, C, D and E	
Number of males/females from A	220/1800
Sire replacement and growth rate	0.5065 0.072
Dam replacement and growth rate	0.30 0.098
Number of males/females from B	160/1,100
Sire replacement and growth rate	0.5851 0.1038
Dam replacement and growth rate	0.30 0.1629
Number of males/females from C	140/1,200
Sire replacement and growth rate	0.5252 0.073
Dam replacement and growth rate	0.30 0.103
Number of males/females from D	120/600
Sire replacement and growth rate	0.6256 0.118
Dam replacement and growth rate	0.30 0.182
Number of males/females from E	100/500
Sire replacement and growth rate	0.5392 0.06
Dam replacement and growth rate	0.30 0.117
Selection	High EBV
Mating system	Random
Number of generations	15
Number of offspring per dam	1
Genome	
Number of chromosomes	29
Number of SNPs	50,000
SNP distribution	Evenly spaced
Number of QTL	800
QTL distribution	Random
MAF of SNPs	0.1
MAF of QTL	0.1
Additive allelic effects for QTL	Gamma
Rate of recurrent mutation	$2.5\times 10^{-5}$

### 2.2 Scenarios

Five scenarios were compared in terms of prediction accuracy of selection candidates in the last generation (15th generation). Scenarios included a reference scenario (Ref. Sc) without genotypic data and scenarios with both genotypic and phenotypic information (Sc. 1 to Sc. 4). In all scenarios, it was assumed that phenotypic and pedigree data were available for the last 15 generations, and animals could be genotyped from the 7th generation onward. In all genomic scenarios, 5,000 animals could be genotyped. Genotyping scenarios differed in the method applied for the selection of 5,000 animals to be genotyped. In Sc. 1, the genotyping strategy was focused on young animals and only male progenies from the pool of selection candidates of generation 15 were selected randomly. In Sc. 2, genotyping was only on males but both young animals and ancestral sires could be genotyped. The criteria for selection of ancestral sires were that a sire should have at least 10 progenies in the population to be selected for genotyping. In Sc. 3, both male and female progenies from generation 15 could be genotyped. In Sc. 4, randomly selected ancestral sires, ancestral dams, and selection candidates (both males and females) were selected to be genotyped. The number of male and female animals with genotypic record in each scenario across breeds is presented in Table 2.

**TABLE 2:** Number of male and female animals with genotyping record in each scenario according to breeds.

^a^Scenarios	Sex	Breeds					
		A	B	C	D	E	Total
Sc. 1	Male progeny	1,349	1,317	1,087	759	488	5,000
Sc. 2	Ancestral sires	621	463	382	305	229	2000
	Male progenies	780	811	647	468	294	3,000
Sc. 3	Male selection candidates	648	670	545	394	239	2,496
	Female selection candidates	654	665	547	402	236	2,504
Sc. 4	Ancestral sires	464	305	303	252	176	1,500
	Ancestral dams	450	386	310	228	126	1,500
	Male selection candidates	249	258	198	167	116	988
	Female selection candidates	259	247	234	175	97	1,012

^a^ Sc. 1: 5,000 randomly selected male progenies from 15th generation were genotyped, Sc. 2: 2000 ancestral sires with more than 10 progenies and 3,000 randomly selected male progenies from 15th generation were genotyped, Sc. 3: 5,000 selection candidates (both males and females) from 15th generation were genotyped, SC 4: randomly selected 1,500 ancestral sires, 1,500 ancestral dams and 2000 selection candidates (both males and females) from 15th generation were genotyped.

**FIGURE 1:**
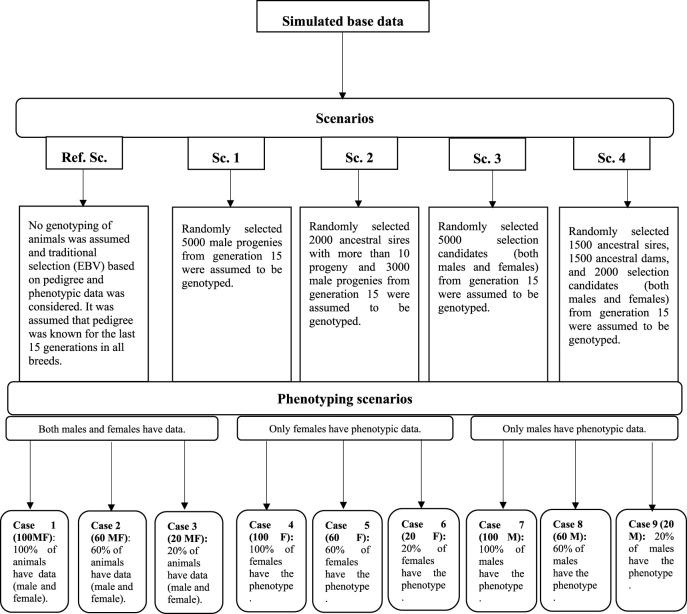
Schematic representation of the genotyping and phenotyping scenarios.

In all scenarios, irrespective of the availability of genotypic data, two other factors were considered to evaluate their impact on the prediction accuracy of the selection candidates. The first factor was the number of phenotypic records which could be collected and used for the genetic evaluation. It was assumed that 20, 60, and 100 percent of the animals could have phenotypic observations (cases 1–3: 100 MF, 60 MF, and 20 MF). This phenotyping scenario was considered to cover a range of traits such as birth weight, where data are collected routinely in practice, and a scarcely recorded trait such as meat quality, where normally 20% of animals in the evaluation would have records available in practice. The second factor was the simulation of a sex-limited trait where either males (e.g., scrotal circumference) (cases 3–6: 100 M, 60 M, and 20 M) or females (cases 6–9: 100 F, 60 F, and 20 F) could have phenotypic records. A schematic representation of the genotyping and phenotyping scenarios is in Figure 1.

### 2.3 Genome architecture

A genome consisting of 29 pairs of chromosomes with a total length of 2,319 cM was simulated. For each animal, single nucleotide polymorphisms (SNPs) markers with the density of 50 K and *n* = 800 QTL were considered. Both SNPs and QTL were selected from the segregating loci of the last generation of the historical population with a Minor Allele Frequency (MAF) of greater than 0.1 and were randomly spaced across the genome. Recurrent mutation rate of $2.5\times 10^{-5}$ for both marker and QTL was considered. The additive allelic effect for each QTL was sampled from gamma distribution with shape parameters equal to 0.4 (Table 1).

### 2.4 Simulation of phenotypes and GEBV

A single trait with a heritability of 0.3 and phenotypic variance of 1.0 was simulated. The True Breeding Values (TBV) for each animal were calculated as follows:

$${TBV}_{k}=\overset{n\;QTL}{\underset{j=1}{\sum }}\beta_{j}.Q_{k_{j}}$$ (1)

Where $\beta_{j}$ is the additive effect of QTL j , $Q_{k_{j}}$ is the QTL genotype at locus j , coded as 0, 1, or 2, as the number of copies of a specified QTL allele is carried by an individual ( k ). The phenotypes $\left(y_{i}\right)$ were simulated by adding residual term sampled as $\varepsilon_{i}∼N\left(0,\sigma_{e}^{2}\right),$ where $\sigma_{e}^{2}$ is the residual variance.

### 2.5 Genetic evaluations

#### 2.5.1 BLUP with unknown parent groups (UPGs)

For the reference scenario, a single-trait BLUP with UPG was used to estimate the breeding values. In this scenario, all five breeds were analyzed together in a multi-breed model. EBVs were estimated based on the model (2):

y=1μ+Xb+Za+ZQs+e (2)

Where y is the vector of simulated phenotypes, µ is the constant average, X is the design matrix connecting records to fixed effects (sex, breed), Z is an incidence matrix relating animals to observations that connects animals to observations; a is the vector of random additive genetic effects; Q is a matrix that contains UPG compounds for all individuals; s is the vector of UPG effects, and e is the vector of random residuals. The trait of interest was considered to be the same across all breeds (i.e., rg = 1). Random effects were assumed to be independent and normally distributed:

$$\begin{array}{ccc} a∼N\left(0,A\sigma_{a}^{2}\right) & and & e∼N\left(0,I\sigma_{e}^{2}\right) \end{array}$$

Where A is the numerator relationship matrix, I is the identity matrix, $\sigma_{a}^{2}$ is the direct additive genetic variance, and $\sigma_{e}^{2}$ is the residual variance. In this model, the EBV was

u=Qs+a (3)

where u was the total EBV, including UPG effects.

#### 2.5.2 ssGBLUP with meta-founder

For genomic scenarios (Sc. 1–Sc. 4), the ssGBLUP with meta-founder was used. In the meta-founder approach, a modified ${\left(H^{\Gamma }\right)}^{-1}$ is substituted for the traditional $H^{-1}$ matrix (Christensen et al., 2015; Legarra et al., 2015)

$${\left(H^{\Gamma }\right)}^{-1}={\left(A^{\Gamma }\right)}^{-1}+\left(\begin{array}{cc} 0 & 0\\ 0 & {\left(G^{\Gamma }\right)}^{-1}-{\left(A_{22}^{\Gamma }\right)}^{-1} \end{array}\right)$$ (4)

Where ${\left(H^{\Gamma }\right)}^{-1}$ is the inverse of the realized relationship matrix with meta-founder, $A^{\Gamma }$ is pedigree relationship matrix formed with a Γ matrix, $A_{22}^{\Gamma }$ is a submatrix of $A^{\Gamma }$ for the genotyped animals, and $G^{\Gamma }$ is genomic relationship matrix with meta-founder constructed as:

$$G^{\Gamma }=\frac{ww^{\prime }}{s}$$ (5)

Where w is the incidence matrix with elements of 1, 0 and −1 for AA, Aa, and aa, respectively; s is the half of the number of markers.

Matrix 𝚪 represents within and across population relationship matrix. The structure of variance-covariance of meta-founder was estimated as $\Gamma =8Cov\left(P\right)$ , according to the method presented by Christensen et al. (2015), where P is a matrix with m columns ( m = total number of meta-founder) and n rows ( n = total number of markers), containing the frequency of the second allele per breed.

The genetic evaluation analysis was performed under the restricted maximum likelihood (REML) approach using an animal model in the BLUPF90 family software (Misztal et al., 2015). The prediction accuracy in each scenario was computed as the correlation between TBV and (G)EBV in the 15th generation.

## 3 Results

In all scenarios, the use of genomic information irrespective of phenotyping strategy, increased the prediction accuracy compared to the Ref. Sc (Table 3). The range of prediction accuracy in Ref. Sc scenario was between 0.14 and 0.34 and for GS scenarios between 0.19 and 0.50. The average prediction accuracy across scenarios were 0.23, 0.31, 0.38, 0.28 and 0.41 for Ref. Sc and Sc. 1 to 4, respectively. Among the GS scenarios, Sc. 4 had the highest accuracy across phenotyping strategies, followed by Sc. 2. In both Sc. 4 and Sc. 2, ancestral animals with contributions to the population (i.e., had some progenies) were genotyped in addition to selection candidates. For scenarios where genotyping was limited to the young selection candidates (Sc. 1 and Sc. 3), prediction accuracy was lower than the scenarios where genotyping was on animals from young and older generations.

**TABLE 3:** Prediction accuracy across scenarios under different phenotyping scenarios.

Cases	Scenarios				
	Ref. Sc	Sc. 1	Sc. 2	Sc. 3	Sc. 4
100 MF	0.34	0.45	0.49	0.39	0.50
60 MF	0.25	0.36	0.43	0.28	0.44
20 MF	0.14	0.21	0.28	0.19	0.34
100 F	0.24	0.35	0.40	0.27	0.41
60 F	0.21	0.30	0.38	0.27	0.35
20 F	0.19	0.24	0.32	0.20	0.38
100 M	0.27	0.36	0.42	0.36	0.45
60 M	0.25	0.30	0.39	0.31	0.42
20 M	0.17	0.26	0.34	0.26	0.39

Details about cases, and scenarios are in Figure 1.

**FIGURE 2:**
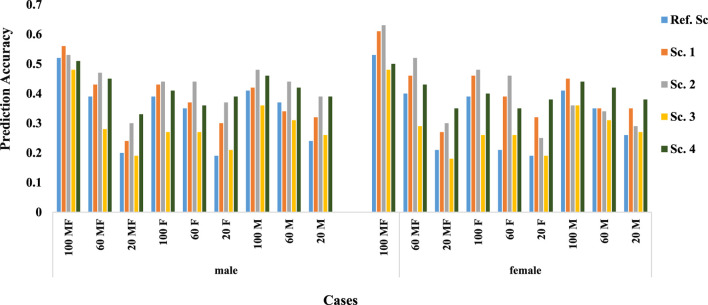
Prediction accuracy in males and females.

Figure 2 shows the prediction accuracy in males and females. Nearly in all scenarios and cases, males were predicted more accurately than females. Even when only 20% of males had a phenotypic record, the accuracy is higher than when 20% of male and female animals or only 20% of female animals had a phenotypic record, which shows the more significant effect of male phenotypic records on the prediction accuracy.

As expected within each scenario, higher percentage of phenotype availability, resulted in higher prediction accuracy. The trend was similar for all scenarios and irrespective of the availability of records on both or either of sex. Results also show that when the trait of interest could be measured on both sex (Cases 100, 60, 20 MF), on average prediction accuracy was higher than when trait was sex-limited. For sex-limited traits, prediction accuracy was similar whether it was measured on males or females, however, mean accuracy were slightly higher when trait of interest was measured on male animals (0.34 in females and 0.36 in males).

Table 4 shows the prediction accuracy for animals without phenotypic records in different cases for selection candidates. The aim would be to realize how genotyping strategy would affect prediction accuracy in animals without records in the last generation. Prediction accuracy in males without records was highest based on Sc. 2 and lowest based on Sc. 3 with an average of 0.41 and 0.24, respectively. Prediction accuracy for females without records was highest in Sc. 4 and Sc. 3 with an average of 0.41 and 0.28, respectively. Note that prediction accuracy for some phenotyping cases is not presented as either all animals had phenotypic records (Case 100 MF) or trait was sex limited (Case 100 M and Case 100 F). (The solution for UPG was similar among breeds and Supplementary Table S1 shows mean solution for UPG each breed across cases.)

**TABLE 4:** Prediction accuracy for animals without phenotypic records in males and females according to cases.

Sex	Cases	Number of animals	Accuracy in scenarios				
			Ref. Sc	Sc. 1	Sc. 2	Sc. 3	Sc. 4
Male	100 MF	—	—	—	—	—	—
	60 MF	5,330	0.39	0.43	0.46	0.28	0.44
	20 MF	5,445	0.20	0.24	0.29	0.19	0.33
	100 F	6,797	0.38	0.43	0.44	0.27	0.41
	60 F	2,719	0.53	0.37	0.43	0.27	0.35
	20 F	5,437	0.18	0.29	0.37	0.20	0.38
	100 M	—	—	—	—	—	—
	60 M	2,719	0.36	0.34	0.43	0.31	0.42
	20 M	5,437	0.24	0.31	0.37	0.26	0.38
Female	100 MF	—	—	—	—	—	—
	60 MF	2,686	0.40	0.45	0.51	0.28	0.43
	20 MF	5,452	0.21	0.26	0.29	0.18	0.35
	100 F	—	—	—	—	—	—
	60 F	2,719	0.20	0.38	0.46	0.25	0.35
	20 F	5,437	0.18	0.31	0.25	0.19	0.37
	100 M	6,797	0.40	0.45	0.36	0.35	0.44
	60 M	2,719	0.35	0.35	0.33	0.31	0.41
	20 M	5,437	0.25	0.34	0.28	0.26	0.38

Note that in 100 MF, 100% of animals have phenotypic records and as a result accuracy was not calculated for this case. Details about cases, and scenarios are in Figure 1.

## 4 Discussion

We investigated the potential of applying GS in beef cattle when the aim was improving prediction accuracy in selection candidates. Four selective genotyping scenarios were compared to traditional pedigree-based evaluation. Results showed fair improvement in prediction accuracy, albeit a limited number of animals being genotyped.

One challenge with applying GS in practice is that there are many selection candidates, and genotyping all of them is often impractical. Genotype scenarios should only genotype small proportions of selected candidates welcomed by breeders because results have shown that significant investments in the genotype of selected candidates are not necessary to take full advantage of the benefits of genomic selection (Pryce and Daetwyler, 2012; Howard et al., 2018). As the results showed that the determination of the genotype of only the selected candidates, regardless of gender, has the lowest prediction accuracy (Sc. 3). Our study investigated the importance and effect of genotyping and phenotyping scenarios on prediction accuracy. The results showed that genotyping of both the selection candidates), and male and female ancestors, could be used to maximize the advantage of genomic selection (Sc. 4). In fact, Sc. 4 confirms the more significant effect of the female genotype than the male genotype and the more significant influence of the ancestral genotype than the selected candidates on the prediction accuracy (because they have more offspring, more information). The lower effect of male genotypes on the accuracy of predictions in this study can be allocated to the effect of the pedigree relationship between individuals. This makes it difficult to make accurate sire selection decisions (Nwogwugwu et al., 2020). In addition, in beef cattle, the offspring are smaller per male and more significant in each female than in dairy cattle. Determining the female genotype in beef cattle can significantly contribute to genetic accuracy and development. As a result, selective genotyping of only part of the selection candidates, males and females of the ancestors, can increase the prediction accuracy. In addition, we can make the most of the benefits of genome selection while saving on genotype costs.

Natural mating of multiple sires is the most common mating system in beef cattle production, despite the management advantages of this mating system, it does not allow for identification the paternity of the progeny (Tonussi et al., 2017). So, because the information of the cows is known, the genotype of females is easier Given that the genotypic data of females are usually more available than males (Mrode, 2019), accuracy can be increased by increasing the genotypic data of females (Tsuruta et al., 2013). Various studies have examined the effect of female genotype on prediction accuracy, including a study using a multi-step method that reported a decrease in accuracy using female genotype (Wiggans et al., 2011). However, in our study using the ssGBLUP approach, accuracy was increased by including the female genotype, and also consistent with the results reported by Tsuruta et al., 2013; Lourenco et al., 2014. In addition, in all genomic scenarios, by examining prediction accuracy in males and females separately, it can be concluded that increasing the genotypic information of females, the prediction accuracy increases (Figure 2).

In practical situation, all animals being evaluated rarely have phenotypic information, and the records are not widely available for traits such as disease and meat quality. To address this issue, we considered different phenotypic scenarios in our simulation. In all studied scenarios, with decreasing phenotypic records, prediction accuracy also decreases, which indicates a direct relationship between phenotypic records and prediction accuracy (Goddard, 2009; Takeda et al., 2020).

Genomic prediction in beef cattle provides accuracy higher than the average of parents based on the pedigree of selected candidates. It can be equivalent to progeny tests based on a maximum of 10 offspring (Garrick, 2011). In addition, ssGBLUP is superior to traditional evaluation methods because ssGBLUP uses phenotypes instead of pseudo-phenotypes and considers the entire population structure for GEBV estimation (Lourenco et al., 2014). This can be used for beef cattle selection in that only a tiny proportion of animals have pedigree and genotype. Estimated breeding value assessment with BLUP depends on the phenotype, parents, and progeny. But the ssGBLUP method is less sensitive to scenarios where animals selectively genotyped and, or genomic preselection exists compared to the multiple-step methods. Hence, ssGBLUP in conventional evaluations is attractive (Masuda et al., 2018). The ssGBLUP method is conceptually and practically simpler than the multiple-step GBLUP method, and in addition, it does not have shortcomings such as bias and loss of information of with few progenies, as well as operational complexity (Christensen and Lund, 2010; Legarra et al., 2014). Therefore, the ssGBLUP method is simpler and applicable to complex models and is generally as accurate as multiple-step methods (VanRaden, 2012; Mehrban et al., 2019). Also, Due to the lower sensitivity of the ssGBLUP method to genotyping scenarios, this method can be used to determine the best genotyping scenario to reduce genotype costs while increasing accuracy (Howard et al., 2018).

In our study, genetic evaluation was based on multiple breeds information. When several breeds are combined in one assessment, there is generally no pedigree information among breeds. As a result, UPG (Quaas, 1988) has been developed to model missing pedigrees and to explain breed differences in multi-breed evaluations(Legarra et al., 2007; VanRaden et al., 2007). However, UPG solutions, when evaluated with the ssGBLUP model, may be biased due to genomic incompatibility ( G ) and pedigree-based relationship ( A ) matrices due to the lack of genotypes of all animals in the pedigree (Misztal and Legarra, 2017; Kudinov et al., 2020). Legarra et al. (2015) developed a meta-founders theory to solve this problem and consider the relationships within and between the founding population. Several studies have reported improved genetic evaluation performance using meta-founder (Bradford et al., 2019; Junqueira et al., 2020). Accordingly, we used the method to account for the genetic level of base animals of each breed in step 4.

The pedigrees used in genetic evaluations may go back to a few base populations thought to be unrelated due to a lack of access to information (Legarra et al., 2015). In addition, information is not available at the beginning of the pedigree, and animals of several generations may have missing pedigree information (Tsuruta et al., 2019). Also, populations are selected, and animals with missing parents are unlikely to be chosen as parents of the next-generation because their breeding value is reduced to zero (Legarra et al., 2015; Kluska et al., 2021). Unknown parent groups and Meta-Founder can be used to calculate missing pedigree and breed structure in multi-breed populations such as beef cattle (Kluska et al., 2021).

We investigated the potential of applying GS in beef cattle when the aim was improving prediction accuracy in selection candidates. Four selective genotyping scenarios were compared to traditional pedigree-based evaluation. Results showed fair improvement in prediction accuracy, albeit a limited number of animals being genotyped. Comparison of GS scenarios revealed that selective genotyping should be on animals from both ancestral and younger generations. In addition, as genetic evaluation in practice cover traits that are expressed on either sex, it is recommended that selective genotyping covers animals from both sexes as well.

## Data Availability

The original contributions presented in the study are included in the article/Supplementary Materials, further inquiries can be directed to the corresponding authors.
