# Supplementary material for: Selective genotyping to implement genomic selection in beef cattle breeding
Source: Front Genet. 2023 Mar 17;14:1083106. doi: 10.3389/fgene.2023.1083106 (PMC10064214; doi:10.3389/fgene.2023.1083106)
Supplement: Supplementary file 1 [file DataSheet1.pdf]

Supplementary Material

1 Supplementary Figures and Tables

Table 1. Parameters of the simulation process

**Population structure**			
**Step 1: Historical generations (HG)**			
Number of generations	phase 1	1000	
Size		1000	
Number of generations	phase 2	200	
Size		2020	
**Step 2: Expanded generations (EG)**			
Number of founder males from HG		100	
Number of founder females from HG		100	
Number of generations		8	
Number of offspring per dam		5	
Selection and mating		Random	
**Step 3: Breed formation (BF)**			
Number of males/females from BF for all 5 breeds		100/100	
Number of generations		30	
Number of offspring per dam		2	
Selection and mating		Random	
**Step 4: Breeds A, B, C, D and E**			
Number of males/females from A		220/1800	
Sire replacement and growth rate		0.5065	0.072
Dam replacement and growth rate		0.30	0.098
Number of males/females from B		160/1100	
Sire replacement and growth rate		0.5851	0.1038
Dam replacement and growth rate		0.30	0.1629
Number of males/females from C		140/1200	
Sire replacement and growth rate		0.5252	0.073
Dam replacement and growth rate		0.30	0.103
Number of males/females from D		120/600	
Sire replacement and growth rate		0.6256	0.118
Dam replacement and growth rate		0.30	0.182

Number of males/females from E	100/500
Sire replacement and growth rate	0.5392 0.06
Dam replacement and growth rate	0.30 0.117
Selection	High EBV
Mating system	Random
Number of generations	15
Number of offspring per dam	1
**Genome**	
Number of chromosomes	29
Number of SNPs	50000
SNP distribution	Evenly spaced
Number of QTL	800
QTL distribution	Random
MAF of SNPs	0.1
MAF of QTL	0.1
Additive allelic effects for QTL	Gamma
Rate of recurrent mutation	2.5×10^{-5}

Table 2. Number of male and female animals with genotyping record in each scenario according to breeds

#Scenarios	Sex	Breeds					
		A	B	C	D	E	Total
Sc. 1	Male progeny	1349	1317	1087	759	488	5000
Sc. 2	Ancestral sires	621	463	382	305	229	2000
	Male progenies	780	811	647	468	294	3000
Sc. 3	Male selection candidates	648	670	545	394	239	2496
	Female selection candidates	654	665	547	402	236	2504
Sc. 4	Ancestral sires	464	305	303	252	176	1500
	Ancestral dams	450	386	310	228	126	1500
	Male selection candidates	249	258	198	167	116	988
	Female selection candidates	259	247	234	175	97	1012

Sc. 1: 5000 randomly selected male progenies from 15th generation were genotyped, Sc. 2: 2000 ancestral sires with more than 10 progenies and 3000 randomly selected male progenies from 15th generation were genotyped, Sc. 3: 5000 selection candidates (both males and females) from 15th generation were genotyped, SC 4: randomly selected 1500 ancestral sires, 1500 ancestral dams and 2000 selection candidates (both males and females) from 15th generation were genotyped.

1.1 Supplementary Figures

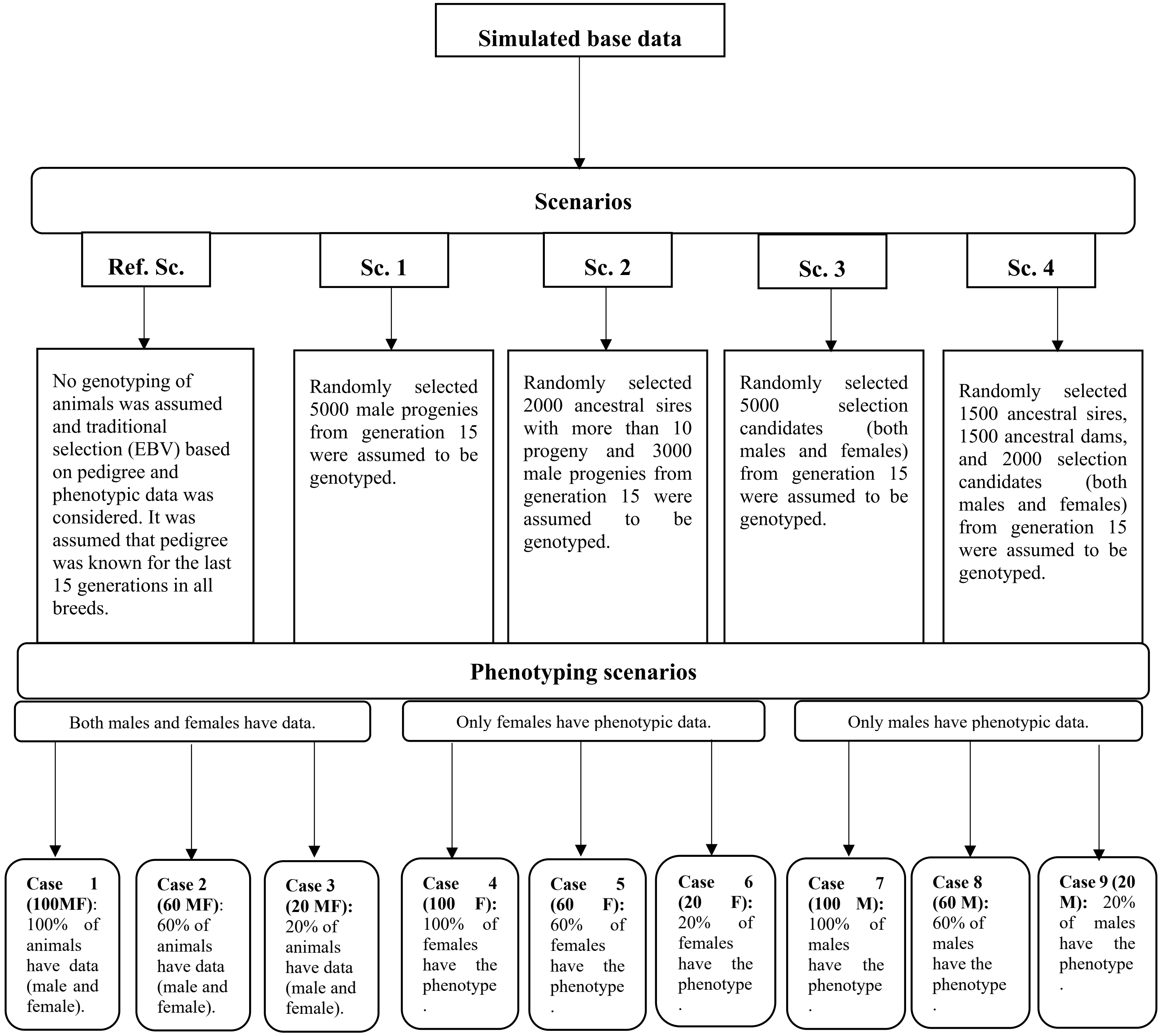

Figure 1. Schematic representation of the genotyping and phenotyping scenarios.

Supplementary Figure 1. A schematic representation of the genotyping and phenotyping scenarios is in Figure 1.

Table 3. Prediction accuracy across scenarios under different phenotyping scenarios.

Cases	Scenarios				
	**Ref. Sc.**	**Sc. 1**	**Sc. 2**	**Sc. 3**	**Sc. 4**
**100 MF**	0.34	0.45	0.49	0.39	0.50
**60 MF**	0.25	0.36	0.43	0.28	0.44
**20 MF**	0.14	0.21	0.28	0.19	0.34
**100 F**	0.24	0.35	0.40	0.27	0.41
**60 F**	0.21	0.30	0.38	0.27	0.35
**20 F**	0.19	0.24	0.32	0.20	0.38
**100 M**	0.27	0.36	0.42	0.36	0.45
**60 M**	0.25	0.30	0.39	0.31	0.42
**20 M**	0.17	0.26	0.34	0.26	0.39

Details about cases, and scenarios are in Figure 1.

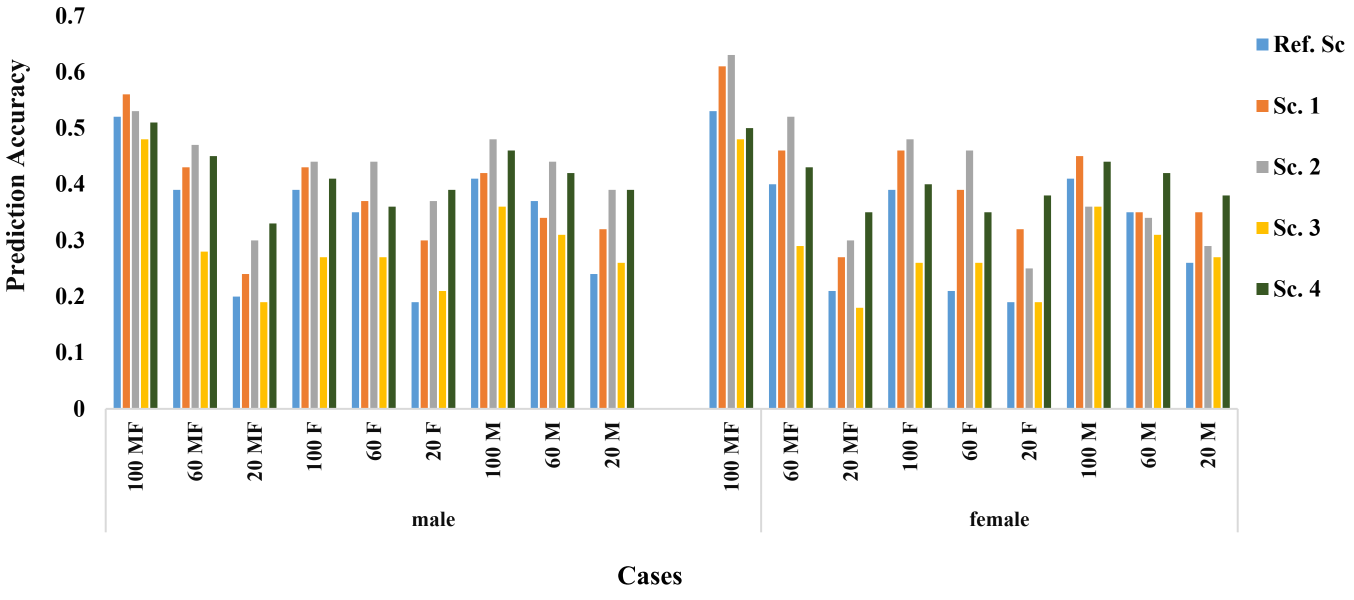

Figure 2. Prediction accuracy in males and females.

Table 4. Prediction accuracy for animals without phenotypic records in males and females according to cases.

Sex	Cases	Number of animals	Accuracy in scenarios				
			Ref. Sc.	Sc. 1	Sc. 2	Sc. 3	Sc. 4
Male	100 MF	-	-	-	-	-	-
	60 MF	5330	0.39	0.43	0.46	0.28	0.44
	20 MF	5445	0.20	0.24	0.29	0.19	0.33
	100 F	6797	0.38	0.43	0.44	0.27	0.41
	60 F	2719	0.53	0.37	0.43	0.27	0.35
	20 F	5437	0.18	0.29	0.37	0.20	0.38
	100 M	-	-	-	-	-	-
	60 M	2719	0.36	0.34	0.43	0.31	0.42
	20 M	5437	0.24	0.31	0.37	0.26	0.38
Female	100 MF	-	-	-	-	-	-
	60 MF	2686	0.40	0.45	0.51	0.28	0.43
	20 MF	5452	0.21	0.26	0.29	0.18	0.35
	100 F	-	-	-	-	-	-
	60 F	2719	0.20	0.38	0.46	0.25	0.35
	20 F	5437	0.18	0.31	0.25	0.19	0.37
	100 M	6797	0.40	0.45	0.36	0.35	0.44
	60 M	2719	0.35	0.35	0.33	0.31	0.41
	20 M	5437	0.25	0.34	0.28	0.26	0.38

Note that in 100 MF, 100% of animals have phenotypic records and as a result accuracy was not calculated for this case. Details about cases, and scenarios are in Figure 1.

Supplementary table. Mean solutions UPG for each breed across cases.

Scenarios	Breeds				
	A	B	C	D	E
Sc.1	-0.061	-0.043	-0.082	-0.028	-0.057
Sc. 2	0.049	0.134	0.039	0.108	0.010
Sc. 3	-0.031	-0.012	0.027	0.015	0.023
Sc. 4	0.301	0.429	0.329	0.333	0.420

Details about cases, and scenarios are in Figure 1.
